# Harnessing biological synthesis: Zinc oxide nanoparticles for plant biotic stress management

**DOI:** 10.3389/fchem.2024.1432469

**Published:** 2024-07-11

**Authors:** Naveen Verma, Priya Kaushal, Amanpreet K. Sidhu

**Affiliations:** ^1^ Department of Biotechnology, Khalsa College, Amritsar, India; ^2^ Department of Biotechnology, Guru Nanak Dev University, Amritsar, India

**Keywords:** zinc oxide nanoparticle, green synthesis, antimicrobial activity, biotic stress, agriculture, toxicity

## Abstract

Crop growth and yield are negatively impacted by increased biotic stress in the agricultural sector due to increasing global warming and changing climatic patterns. The host plant’s machinery is exploited by biotic stress, which is caused by organisms like bacteria, fungi, viruses, insects, nematodes, and mites. This results in nutrient deprivation, increased reactive oxygen species and disturbances in physiological, morphological, and molecular processes. Although used widely, conventional disease management strategies like breeding, intercropping, and chemical fertilizers have drawbacks in terms of time commitment and environmental impact. An environmentally beneficial substitute is offered by the developing field of nanotechnology, where nanoparticles such as zinc oxide are gaining popularity due to their potential applications as antimicrobials and nano-fertilizers. This review delves into the biological synthesis of ZnO nanoparticles employing plants and microbes, function of ZnO nanoparticles in biotic stress mitigation, elucidating their effectiveness and toxicological implications in agricultural. This study supports a cautious approach, stressing the prudent application of ZnO nanoparticles to avoid possible toxicity, in line with the larger global agenda to end hunger, guarantee food security, and advance sustainable agriculture.

## 1 Introduction

Extensive global warming and change in climate patterns increase the incidences of biotic stress in crops, which negatively impact their growth and yield. The term “biotic stress” describes the detrimental effect of bacteria, fungi, viruses, insects, nematodes, and mites, etc. on the plant growth, development, and yield (Pandey et al., 2017; Masri and Kiss, 2023). These agents generally utilized the host plant machinery for their growth and survival, which in turn causes nutrients deprivation, increases ROS, disturbs the physiological, morphological and molecular functioning of the plants and ultimately lead to their death (Gull et al., 2019; Chaudhary et al., 2022). Moreover, during the postharvest processing of grains and fruits, pathogenic infections in the plants are the primary source of significant economic loss. The projected yearly loss of crop yield due to pests and pathogens is estimated as US$220billion (Singh et al., 2023). By 2050, crop productivity is expected to rise by 60%–100%. In order to feed the world’s anticipated 9.7 billion people different disease management techniques like breeding, intercropping, development of stress resilient varieties, and usage of chemical fertilizers are widely employed to mitigate the biotic stress (Lau et al., 2022; Mohan et al., 2023). However, these approaches are time-consuming and extensive application of chemical pesticides, insecticides and herbicides increase the problem of pathogens resistance, soil degradation and also effects human health (Lau et al., 2022). The United Nations has embraced a mandate encompassing 17 Sustainable Development Goals (SDGs) directed towards fostering global sustainable development, with a fundamental aim to ensure worldwide peace, prosperity, and advancement. Goal 2 of this collective initiative seeks to eliminate hunger, establish food security, enhance nutritional standards, and advocate for sustainable agriculture practices (https://sdgs.un.org/goals).

**FIGURE 1:**
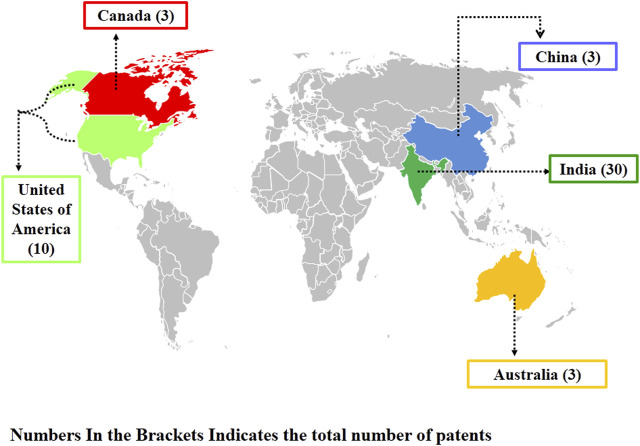
Worldwide distribution of Patents in the field of utilization of nanoparticles as “Nanofertilizers”.

Nanotechnology branch has emerged as an ecologically friendly alternative for conventional agricultural chemicals/pesticides/herbicides (Hajong et al., 2019; Khan et al., 2021a; Shams et al., 2023). Different nanoparticles like silver, copper oxide, silicon oxide, nano-calcite, magnesium oxide, zinc oxide, and titanium dioxide, etc. have been reported as potential nano-fertilizers, antibacterial, and antifungal nanomaterials (Khan et al., 2021b; Mohan et al., 2023; Tortella et al., 2023). The main mechanism by which these nanoparticles kills the pathogenic microorganisms includes inhibition of biofilm, cell membrane damage, formation of reactive oxygen species (ROS) and reactive nitrogen species (RNS) free radicals (peroxides, superoxide, hydroperoxyl, hydroxyl radical, singlet oxygen, nitric oxide, peroxynitrite). Once the ROS and RNS get accumulated in the microbial cell it will damage the structure and functioning of biological macromolecules like proteins, nucleic acids and lipids (Alavi and Yarani, 2023). In the era of green technology, nanoparticles as nano-fertilizers are an emerging field. The patent analysis data by selecting “Any field” in “Field” option and using “Nanofertilizers” keywords (https://patentscope.wipo.int/search/en/result.jsf?_vid=P11-LS5W5W-53175) revealed that India is leading in this area (Figure 1), but patents related to the application of zinc oxide nanoparticles as fertilizers/pesticides are limited. The annual production of ZnO NPs is estimated to be between 550 and 33,400 tonnes, and these are the third most used metal containing nanomaterial (Faizan et al., 2021). Keeping this in mind, nowadays, researchers from all over the world have been shelling out serious attention to ZnO NPs because of their nano-size, ionic nature, crystal structure, dissolution rate, ability to cross plant cell-wall, and large surface area-to- volume ratio (Miri et al., 2019; Verma et al., 2023). These nano-sized ZnO NPs can easily interacts with bacterial/fungal cell surface and core, and thereby enters the pathogens and exhibits antimicrobial activity. The size, shape, surface to volume ratio, and quantity of oxygen vacancy sites of ZnO NPs all directly affects their antimicrobial efficacy (Sirelkhatim et al., 2015; Happy et al., 2019; Ogunyemi et al., 2019). Therefore, in the present epoch of nanotechnology, the utilization of ZnO NPs have been extensively investigated.

No doubt, the ZnO NPs have broad application in the disease management but from the literature analysis it has been observed that research in this area is very limited. Researcher faced many challenges for utilization of these nanoparticles for different plants. For example, it is unclear how these nanoparticles work under biotic stress for various plants, and the best synthesis method for ZnO NPs, optimum size and shape, and most important best concentration for better results without causing any toxicity to plants. Keeping all these blind spot in view, this review highlights various important studies for better utilization of ZnO NPs. Firstly, we described the biological methods for the synthesis of ZnO NPs. Secondly, we have elaborated their role as antimicrobial agent in biotic stress mitigation in different crop plants. Lastly, an opinion for the utilization of these nanoparticles in a strict manner to avoid the toxicity of these nanoparticles for plants health is also highlighted. This study is also contributing important role in zero hunger goal of SDGs for better tomorrow by enhancing the yield and growth of crop by eliminating the biotic stress.

## 2 Synthesis of ZnO NPs

Generally, the synthesis of ZnO NPs is carried out by chemical, physical, and biological techniques. Chemical approach involves extensive utilization of co-precipitation, precipitation, water-oil microemulsion, hydrothermal, cellulothermal, sonochemical, sol-gel processing, colloidal, and polyol methods. Furthermore, physical approach is based upon thermal evaporation, ultrasonic irradiation, laser ablation, and arc plasma method (Al-darwesh et al., 2024). However, all these techniques are costly, shows lack of repeatability and dependability, based upon additional supplementation of capping and reducing agents, require toxic chemicals, high temperature and pressure for the process (Balogun et al., 2024). Moreover, toxic chemicals used in these processes sometimes resides inside the synthesized nanoparticles, that in turn reduces the activity of NPs (Kharde et al., 2024).

In order to overcome these drawbacks over the past 10 years, there has been an apparent interest in the biological approach for the synthesis of ZnO NPs. The main reason for the successful utilization of this method is based upon the lower energy and cost requirements for the process, lack of usage of hazardous chemicals, renewable feedstocks, and less release of noxious secondary waste (Mirzaei and Darroudi, 2017; Bandeira et al., 2020). In this context, the following section provides the comprehensive details regarding the green synthesis of ZnO NPs using plant, bacteria, fungi, and algal extracts.

### 2.1 Plant extract

**FIGURE 2:**
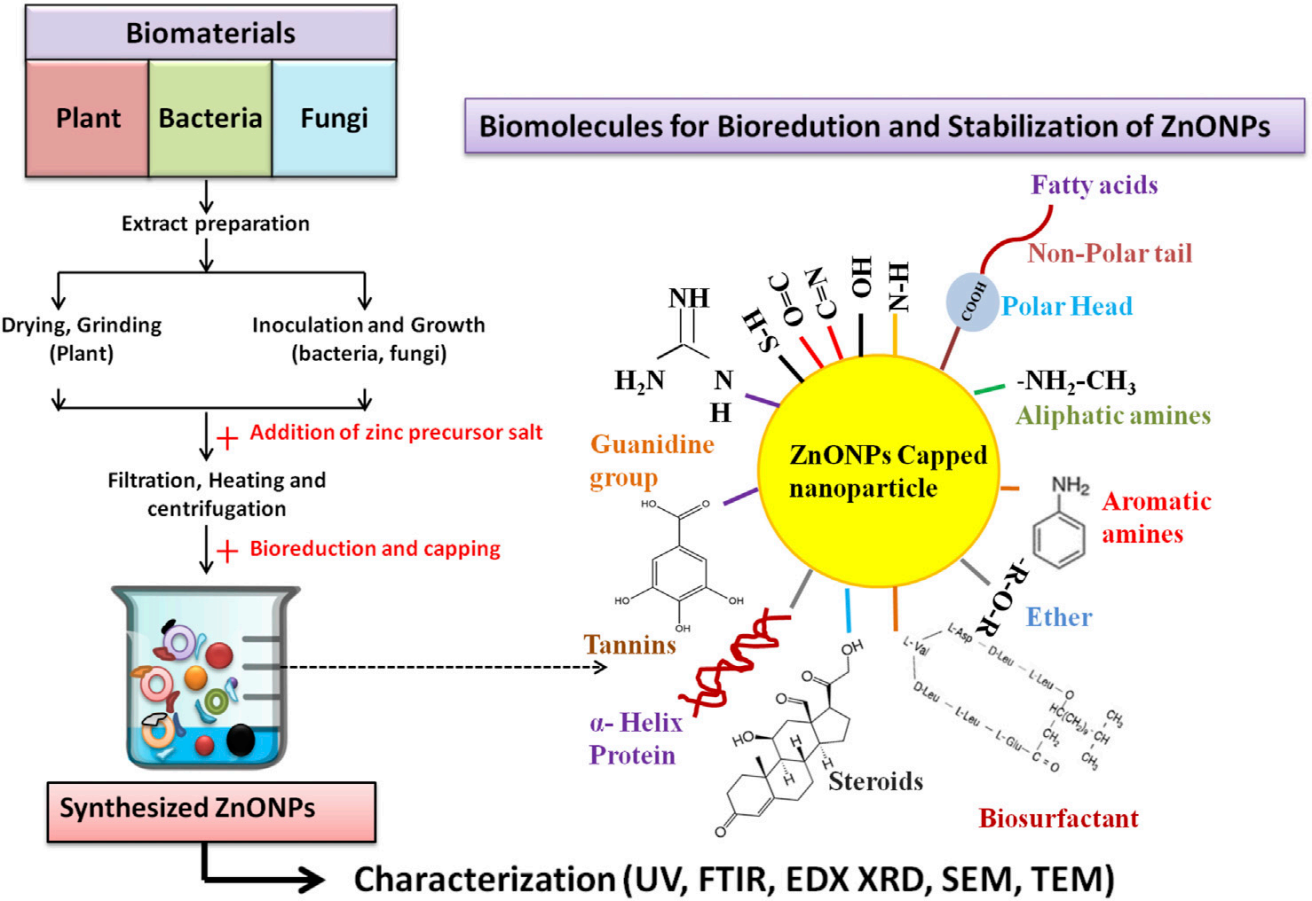
Schematic representation of green synthesis of ZnO NPs by plant, bacteria and fungi. Bold arrows (→) indicates the different steps involved in the synthesis process; (→) dotted arrow indicates the biological reduction, capping and stabilization of synthesized ZnO NPs by natural biomolecules present in the extracts.

In the scientific research community, plants offer a cheap, safe, biocompatible, and readily available green material for the synthesis of various types of metallic nanoparticles. Different plant parts like leaves, roots, flowers, stem, and seed husk are rich deposits of natural reducing and capping agents like carbohydrates, amino acids, vitamins, lipids, proteins, secondary metabolites (steroids, flavonoids, coumarins, terpenoids, tannins) that plays an important role in the fabrication, and stabilization of synthesized nanoparticles (Priya et al., 2021; Sidhu et al., 2022) (Table 1; Figure 2].

**TABLE 1:** Green synthesis of ZnO NPs by using plants, bacteria and fungi.

Biomaterial	Zinc precursor salt	Reaction set up	Size of ZnO NPs	Shape of ZnO NPs	References
Plants					
*Sesbania grandiflora* (Leaf extract)	0.01M zinc acetate solution	Yellow color paste of 2 mL of leaf extract+ 50 mL Zinc acetate solution + final heating at 350°C for 3 h	45 nm	Spherical	Selvaraj et al. (2020)
*Matrocaria chamomilla* (flower extract), *Olea europaea* (leaf extract), *Lycopersicon esculentum* M. (fruit extract)	1M zinc oxide	1:1 ratio of zinc oxide + Plant extract + continuous stirring at 100 rpm for 4 h + centrifugation at 10,000 g for 20min. The resultant NPs pellet was collected and freeze dried	51.2 ± 3.2 nm; 41.0 ± 2.0 nm; 51.6 ± 3.6 nm	Crystalline	Ogunyemi et al. (2019)
*Dolichos Lablab* L. (Leaf extract)	2.73mmol zinc acetate dihydrate	10 mL leaf extract+ 0.5 g zinc acetate+ 10mmole NaOH+ 80 mL Mili-Q subjected to reflux at 80°C under magnetic stirring for 1 h	7–49 nm	Hexagonal and triangular	Kahsay et al. (2019)
*Citrus sinensis* (Fruit peel extract)	Zinc nitrate	42.5 mL of peel extract+ 2 g of zinc nitrate + stirring for 60 min + heating at 60°C for 1 h + drying at 150°C and heat treatment at 400°C for 1 h	Randomly distributed size, i.e. 35–60 nm, 70–100 nm and 200–300 nm	Spherical	Thi et al. (2020)
*Eucalyptus globules* (Leaf extract)	1mM zinc nitrate hexahydrate	1:2 ratio of zinc nitrate + Plant extract + continuous stirring at 150°C for 2–3 h + centrifugation of solid product at 6000 rpm for 10 min + washed and dried at 80°C for 5–6 h	52–70 nm	Spherical and few particles were elongated	Ahmad et al. (2020)
*Sambucus ebulus* (Leaf extract)	1M zinc acetate dihydrate	2 mL of extract+20 mL of zinc solution + continuous stirring at 80°C for 2 h + centrifugation at 7168 *g* for 15 min + drying of precipitates at 60°C + calcination at 450°C for 2 h	25–30 nm	Spherical	Alamdari et al. (2020)
*Melia azedarach* (Leaf extract)	0.006M zinc nitrate hexahydrate	75 mL of zinc nitrate solution+25 mL of plant extract subjected to continuous stirring at 50°C for 24 h; followed by calcination at 200°C for 30min	33–96 nm	Hexagonal and Spherical	Dhandapani et al. (2020)
*Cassia fistula*, *Melia azedarach* (Leaf extract)	0.01M zinc acetate dihydrate	95 mL of zinc acetate solution+5 mL of each plant extract were separately incubated at 70°C with shaking at 150 rpm for 1 h. Powdery precipitate was centrifuged at 3000 rpm for 30min	68.1 nm and 3.62 nm	Spherical	Naseer et al. (2020)
*Beta vulgaris* (BE1), *Cinnamomum tamala* (VA1), *Cinnamomum verum* (PA1)*, Brassica oleracea* var. *Italica* (BR1) (Plant extract)	Zinc nitrate	50 mL of each extract+5 g of zinc nitrate was boiled and the resultant white paste was heated in a muffle furnace at 400°C for 2 h	BE1 = 20 ± 2 nm VA1 = 30 ± 2 nm PA1 = 46 ± 2 nm BR1 = 47 ± 2 nm	BE1 = Spherical VE1 = Rod shaped PA1 = Spherical BR1 = Spherical	Pillai et al. (2020)
*Deverra tortuosa* (Plant extract)	Zinc nitrate hexahydrate	25 mL of plant extract+2.5 g of zinc nitrate subjected to heating on magnetic stirrer for 1 h; then mixture was kept in a hot air oven at 60°C for overnight. Creamy paste obtained was washed with 3:1 distilled water: ethanol	9.26–31.18 nm	Hexagonal	Selim et al. (2020)
*Salvia officinalis* L. (Leaf extract)	0.2M zinc nitrate	50 mL of leaf extract+50 mL of zinc nitrate solution was subjected to stirring at 50°C for 2 h +2.0M NaOH solution was added + centrifugation at 10,000 rpm for 20 min. In the final step material was heated in the muffle furnace for 2 h at 400°C	26.14 ± 2.46 nm	-	Abomuti et al. (2021)
*Myristica fragrans* (Fruit extract)	Zinc acetate dihydrate	100 mL of extract+6.0 g of zinc acetate kept on the magnetic stirrer at 60°C for 2 h; followed by centrifugation at 10,000 rpm for 10 min. The pellet was dried, calcined for 2 h at 400°C	43.3–83.1 nm	Spherical and Hexagonal	Faisal et al. (2021)
*Cayratia pedata* (Leaf extract)	0.1mM zinc nitrate	5 mL of zinc nitrate + leaf extract subjected to stirring at 65°C for 20 min; followed by calcination at 400°C for 2 h	52.24 nm	-	Jayachandran et al. (2021)
*Justicia adhatoda* (Leaf extract)	Zinc sulfate; zinc nitrate; zinc acetate dihydrate	40 mL of different zinc salts+ 10 mL leaf extract+2M NaOH followed by centrifugation at 3000 rpm for 15 min +washing + drying at 150°C in the hot air oven	15–20 nm	Orthogonal/nanorod	Pachaiappan et al. (2021)
*Cassia auriculata* (Leaf extract)	0.1M zinc acetate	5 mL of leaf extract+100 mL of zinc acetate+2 g NaOH subjected to continuous stirring for 2 h + centrifugation at 6000 rpm for 30 min + boiling for 1 h + heating at furnace at 400°C	20–30 nm	Nano Rod and Nano flower	Ramesh et al. (2021)
*Phoenix roebelenii* (Leaf extract)	Zinc nitrate hexahydrate	6.0 g zinc nitrate +100 mL of extract + continuous stirring for 20 h + centrifugation + Precipitate drying at 100°C for 2 h + calcination at 500°C for 2 h in open air tubular furnace	15 ± 0.37 nm	Spherical	Aldeen et al. (2022)
*Evolvulus alsinoides* (Leaf extract)	91mM zinc acetate	10 mL of extract+ 50 mL of zinc acetate solution + continuous stirring + centrifugation at 10,000 rpm for 10min + dried at 40–50°C	100 nm	Spherical	Yadav et al. (2024)
*Carica papaya* (Fruit extract)	0.1M zinc nitrate hexahydrate	10 mL of extract +90 mL of zinc nitrate + stirring at 60°C for 1 h + centrifugation at 16,000 rpm for 15min + heating at 500°C for 2 h in the furnace	170 nm	Spherical and Hexagonal	Easmin et al. (2024)
*Cymodocea serrulata* (Leaf extract)	10mM zinc nitrate	100 mL of zinc nitrate solution + 5–10 mL of leaf extract + continuous stirring + centrifugation at 12,000 rpm + drying of pellet in the hot air oven at 65°C for 24 h	Less than 100 nm	-	Harshitha et al. (2024)
*Allium cepa* L. (Peel extract)	2mM zinc acetate	20 mL peel extract +100 mL of zinc acetate solution + stirring + centrifugation at 700 rpm for 5min + drying + calcination at 500°C for 2 h	49 nm to below 100 nm	Hexagonal	Islam et al. (2024)
*Cissus quadrangularis* L. (Stem extract)	1mM zinc acetate	50 mL zinc acetate +20 mL NaOH +25 mL stem extract + Incubation for 1 h + continuous stirring for 3 h	88.7–182 nm	Spherical	Nazneen and Sultana (2024)
*Allium sativum* (Plant extract)	Zinc nitrate	20 mL plant extract + 2 mL zinc nitrate + heating in the water bath at 80°C for 8 h + calcination for 2 h at 450°C	-	Aggregated irregular	Vithalani and Bhatt (2024)
*Aloe vera* (Leaf extract)	0.6M Zinc acetate dihydrate	1:1 ratio of zinc acetate and leaf extract + stirring for 30min + Heating at high pressure bioreactor at 150°C for 6 h + centrifugation at 4500rpm for 15min + drying at 80°C in a vacuum oven for 8 h	30 nm	Irregular	Wu et al. (2024)
**Bacteria**					
*Sphingobacterium thalpophilum*	1mM zinc nitrate	100 mL cell free supernatant +100 mL zinc nitrate solution + stirring at room temperature for 24 h + drying at 120°C + annealing at 700°C for 5 h	112 nm	Triangle chips like	Rajabairavi et al. (2017)
*Nostoc* sp. EA03	0.02M zinc acetate dihydrate	100 μL cell free extract +50 mL zinc acetate + stirring for 10min + 0.1M NaOH + vigorous stirring for 2 h + centrifugation at 5000rpm for 15min + drying of pellet in a vacuum oven at 60°C for 2 h	60 nm	Star shaped	Ebadi et al. (2019)
*Pseudomonas putida* MC2989	Zinc nitrate	100 mg zinc nitrate + diluted culture + incubation at 37°C at 150rpm for 24 h + centrifugation at 5000rpm for 15min + drying in the hot air furnace at 400°C for 2 h	44.5 nm	Hexagonal	Jayabalan et al. (2019)
*Lactococcus lactis* NCDO1281(T) and *Bacillus* sp. PTCC 1538	0.1M zinc nitrate hexahydrate	50 mL of bacteria culture + zinc nitrate solution + heating in the water bath at 70–80°C for 5–10min + incubation at 37°C at 130rpm for 24 h + centrifugation of pellet at 34000rpm + drying at 40°C in the oven	55 nm (T) 99 nm (PTCC 1538)	Spherical (T); Nano rods (PTCC1538)	Mahdi et al. (2021)
*Pseudomonas aeruginosa* NMj15	2mM Zinc acetate dihydrate	100 mL of cell free supernatant + zinc acetate solution + incubation at 35 ± 2°C for 24 h with shaking + Oven drying of precipitates at 150°C for 24 h	14.95 ± 3.5 nm	Spherical	Abdo et al. (2021)
*Paraclostridium benzoelyticum* strain 5,610	0.1M zinc nitrate	100 mL of bacterial culture + zinc nitrate solution + heating at 80°C for 10min + incubation of white precipitates for 24 h + centrifugation at 14,000 rpm for 10min + drying at 120°C	50 nm	Spherical and Rectangular	Faisal et al. (2022)
*Bacillus foraminis*	0.1M zinc sulfate	50 mL of zinc sulfate +50 mL of culture filtrate + shaking and heating at 40°C for 15min + incubation in the microwave for 1–2min + chilling for 1 h + centrifugation at 3000 rpm for 10min + drying of pellet in the oven at 40°C for 8 h	16–25 nm	Polycrystalline	EL-GHWAS (2022)
*Priestia megaterium*	1mM zinc sulfate heptahydrate	50 mL of zinc sulfate + culture filtrate + incubation at 30°C, 150 rpm for 24 h + centrifugation at 10, 000 rpm for 10min	5.77–13.9 nm	Semi-spheres	Ashour and Abd- Elhalim (2024)
*Bacillus paramycoides*	4mM zinc sulfate	20 mL of cell free supernatant +20 mL of zinc sulfate solution +0.2% polyvinylpyrollidone (PVP) + 0.2% isopropanol +15 kGy dose of gamma radiations	4.37 nm	Hexagonal	El-Refai et al. (2024)
**Fungi**					
*Aspergillus fumigatus* JCF	1mM zinc sulfate	10 mL filtrate +10 mL zinc sulfate solution + incubation in the shaker at 150rpm at 32°C for 72 h + centrifugation of aggregates at 10,000 rpm for 10min + lyophilization	60–80 nm	Spherical	Rajan et al. (2016)
*Aspergillus niger*	1mM zinc acetate	Cell free filtrate +50 mL of zinc acetate + continuous stirring for 24 h + drying of precipitates at 150°C for 48 h	80–110 nm	Rod and cluster shape	Gao et al. (2019)
*Xylaria acuta*	Zinc nitrate hexahydrate	2–10 mL of fungal extract + 1 g of zinc nitrate + continuous stirring + heating at 400°C ± 10°C in the muffle furnace + calcination at 700°C for 2 h	40–55 nm	Hexagonal	Sumanth et al. (2020)
*Alternaria tenuissima*	Zinc sulfate heptahydrate	100 mL of zinc sulfate solution +100 mL of cell free culture + vigorous stirring for 20min + ultracentrifugation for 20 min at 20, 000 rev min^-1^ + drying at 50°C in the oven	15.62 ± 4.51 nm	Spherical	Abdelhakim et al. (2020)
*Phanerochaete chrysosporium*	0.1M Zinc sulfate heptahydrate	100 mL of cell free filtrate +100 mL of zinc sulfate solution + incubation at 27°C for 24 h + drying at 100°C for 24 h	5–200 nm	Hexagonal	Sharma et al. (2021)
*Aspergillus niger*	Zinc acetate	1 g of cell free filtrate dissolved in 100 mL ethanol +10 g of zinc acetate dissolved in 1L distilled water + heating in the water bath for 30min + addition of 0.1M NaOH + Freeze drying + centrifugation at 4000rpm for 10min	23.97 ± 2.29 nm	Spherical	Abdelkader et al. (2022)

Selvaraj et al. (2020) highlighted the synthesis of ZnO NPs by using aqueous leaf extract of *Sesbania grandiflora*. The fourier transform infrared spectroscopy (FTIR) spectra of synthesized NPs showed broad peaks in the region at 3,441 and 3,105 cm^−1^, 1,611 and 1,555 cm^−1^, and 1,440 and 1,392 cm^−1^ that mainly corresponds to the vibrations of–OH, C=O, and -C-H functional groups. This clearly indicates the bioreduction of ZnO NPs by the biomolecules present in the fresh leaf extract. In terms of the antimicrobial efficacy of the synthesized ZnO NPs, leaf extract based NPs showed significant inhibition of *Pseudomonas aeruginosa* and *Staphylococcus aureus* pathogens growth as compared to chemically synthesized ZnO NPs. Furthermore, Ogunyemi et al. (2019) team synthesized ZnO NPs by using the three different plant extracts, i.e., *Matrocaria chamomilla* L. flowers, *Olea europaea* leaves, and *Lycopersicon esculentum* M. fruit. The results of FTIR spectra of ZnO NPs synthesized by three types of extracts showed broad bands of O-H, CH_3_, C=O, C-O-H, and C-N vibrations confirming the presence of terpenes, flavonoids, tannins, glycosides, and alkaloids in the extract that acts as reducing and capping agents for the synthesis of NPs. The ZnO NPs were also effective in retarding the growth of *Xanthomonas oryzae* pv. *oryzae* (Xoo) strain GZ 0003 by inhibiting biofilm formation, bacterial cell membrane, and swimming motility, that in turn helps in controlling bacterial blight disease. In the same year, Kahsay et al. (2019) reported the facile, green strategy for the synthesis of ZnO NPs by using aqueous leaf extract of *Dolichos Lablab* L. The possible functional groups involved in the reduction of ZnO NPs were confirmed by FTIR analysis. –OH, C-H, C=O, C-O, N-O stretching vibrations clearly indicates that alcohol or phenol, alkane, amide, carboxylic, ester groups from the alkaloid, phenol, flavonoid, amino acid, protein, terpenoid and saponin phyto-constituents of the extract were responsible for the stabilization of ZnO NPs. Additionally, 2.5–10 mg/mL concentration of synthesized ZnO NPs also inhibits *Bacillus pumilus* and *Sphingonomas paucimobilis* pathogen effectively as same as standard ciprofloxacin antibiotic. Interestingly, Thi et al. (2020) shed lights on the efficient way of synthesis of ZnO NPs by using *Citrus sinensis* aqueous fruit peel extract. The FTIR analysis of the synthesized ZnO NPs at different annealing temperatures (300°C–900°C) showed common C=C, and C=O functional groups. Further, it has been observed that 0.025 mg/mL concentration of ZnO NPs were effective in producing ROS in the water suspension that in turn excellently retards the activity of *Escherichia coli* and *S. aureus*. Furthermore, research work carried out by Ahmad et al. (2020); Alamdari et al. (2020); Dhandapani et al. (2020); Naseer et al. (2020); Pillai et al. (2020); Selim et al. (2020) provides the biogenic approach for the synthesis of ZnO NPs by using *Eucalyptus globules*, *Sambucus ebulus*, *Melia azedarach*, *Cassia fistula*, *M. azedarach*, *Beta vulgaris*, *Cinnamomum tamala*, *Cinnamomum verum*, *Brassica oleracea* var. *Italica*, *Deverra tortuosa* plants. It has been observed that in the case of *E. globules* leaves extract the FTIR analysis of the synthesized ZnO NPs revealed that presence of C-H groups were mainly associated with the aromandendrenemyrtenal, borneol, camphene, carvacrol, citronellal, citronellyl acetate, cryptone-α-terpenyl acetate essential oil components, which helps in the capping and stabilization of the synthesized NPs. Additionally, flavonoids, protein molecules and other phytochemicals help in the biological reduction of the ZnO NPs.

Similarly, Abomuti et al. (2021); Faisal et al. (2021); Jayachandran et al. (2021); Pachaiappan et al. (2021); Ramesh et al. (2021) also provides attention on the green bio-fabrication of ZnO NPs by using aqueous leaf extract of *Salvia officinalis* L., fruit extracts of *Myristica fragrans*, plant extract of *Cayratia pedata*, leaf extract of *Justicia adhatoda*, and *Cassia auriculata*. A deep analysis of various functional groups involved in the biological synthesis process revealed that rosmarinic acid, chlorogenic acid, ellagic acid, and luteolin 7-glucoside, polysaccharides, pectins, cellulose, alkynes, terpenoids, and flavonoids had a profound effect on the reduction of ZnO NPs. Intriguingly, Aldeen et al. (2022) first time reports the synthesis of ZnO NPs by using aqueous extract of *Phoenix roebelenii* palm leaves. In this work, time dependent FTIR spectral analysis was performed for determining the mechanism of stabilization of ZnO NPs by the biomolecules in the extract. The existence of phenolic compounds such as quercetin 3-O-glucoside, apigenin 6,8-di-C-β-glucopyranoside, luteolin 3′-O-β-glucopyranoside, quercetin, luteolin, apigenin, baicalein, p-hydroxybenzoic acid, caffeic acid and vanillic acid functions both as chelating and capping agents that prevents the aggregation of synthesized ZnO NPs. Recently, Yadav et al. (2024); Easmin et al. (2024); Harshitha et al. (2024); Islam et al. (2024); Nazneen and Sultana, 2024; Vithalani and Bhatt, 2024; Wu et al. (2024) also highlights the biological synthesis of ZnO NPs by using extracts of *Evolvulus alsinoides*, *Carica papaya* peel, *Cymodocea serrulata*, *Allium cepa* L. peel, *Cissus quadrangularis* L. stem, *Allium sativum*, and *Aloe vera* plants. In continuation to the fabrication of the synthesized ZnO NPs, phytochemicals such as squalene, piperine, unsaturated fatty acids, polyphenols, alkaloids, carboxylic acids acts as a bio-reduction agents.

Overall, the presence of natural reducing, capping, fabricating, and stabilizing agents in the plants extracts bypass the toxic chemicals for the synthesis of the ZnO NPs.

### 2.2 Microbes based synthesis of ZnO NPs

Microorganisms based biological synthesis of metallic nanoparticles is considered as an inexpensive, simple, highly productive, and biocompatible strategy (Abdo et al., 2021). Several reports showed that microbes utilizes inherent intracellular and extracellular mechanism for the synthesis of metallic nanoparticles. The presence of metal ion transporters, enzymes, co-enzymes, polysaccharides, and proteins over the microbial cell provide the surface for binding of the metal ions, followed by the reduction of Zn^2+^ by NADH- dependent electron transfer (Mohd Yusof et al., 2019; Sidhu et al., 2022) [Table 1; Figure 2].

Rajabairavi et al. (2017) reported the synthesis of ZnO NPs by using endophytic bacteria *Sphingobacterium thalpophilum*. The results speculates that proteins present in the bacterial culture supernatant helps in the bio-reduction/fabrication of ZnO NPs by electrostatic and hydrophobic interactions. Furthermore, Ebadi et al. (2019); Jayabalan et al. (2019) also demonstrates the green synthesis of ZnO NPs by using cyanobacterium *Nostoc* sp. EA03 and *Pseudomonas putida* culture. FTIR spectrum revealed that the fatty acids, and phenolic OH amino acids (tyrosine, phenylalanine) of the bacterial extracellular proteins contribute in the capping of the ZnO NPs. Mahdi et al. (2021) and his coworkers also elucidate the biosynthesis of ZnO NPs using *Lactococcus lactis* NCDO1281(T) and *Bacillus* sp. PTCC 1538 strains. The characterization of ZnO NPs by both the strains showed that the cell membrane proteins were mainly involved in the synthesis and stabilization of NPs in the culture medium. Moreover, Abdo et al. (2021) also highlighted the green synthesis of ZnO NPs using *P. aeruginosa* NMj15. In this research, the functional groups (C=O, O-H, NH, and SH) of metabolites present in the cell free filtrate of the strain acted as biocatalysts, reducing and stabilizing agents for the synthesis of ZnO NPs. Additionally, Faisal et al. (2022); EL-GHWAS (2022) also presented the ZnO NPs synthesis by using *Paraclostridium benzoelyticum* strain 5,610 and *Bacillus foraminis*. The FTIR analysis findings demonstrated that the carbonyl groups from the amino acids residues of the proteins made a substantial contribution in the capping of the nanoparticles. Recently, Ashour and Abd- Elhalim (2024), El-Refai et al. (2024) synthesized ZnO NPs using *Priestia megaterium* and *Bacillus paramycoides* bacteria. The results point out that the ZnO NPs were coated with the C, N, O, Cl, K, and Na atoms which clearly indicate that enzymes and proteins present in the cell free supernatant help in the reduction of Zn metal ions.

Besides bacteria, fungal extract is also used for the synthesis of ZnO NPs. Rajan et al. (2016) reported the synthesis of ZnO NPs by using fungus *Aspergillus fumigatus* JCF. It has been observed that synthesized ZnO NPs were mainly coated with N-H, C=C, C=O functional groups. All these functional moieties were from the aliphatic, aromatic amino acids, and proteins present in the fungal filtrate. Furthermore, another strain of *Aspergillus*, i.e., *Aspergillus niger* has also been used for the synthesis and bio-fabrication of ZnO NPs (Gao et al., 2019). The results imply that the strong aromatic rings and carboxylic acids of the fungus biomolecules helps in the reduction and capping process of the ZnO NPs. Sumanth et al. (2020) point up the mycogenic synthesis of ZnO NPs by using *Xylaria acuta* extract. The FTIR spectrum showed that the OH groups of hydroxyl and polyphenols were mainly involved in the utilization of ZnO NPs. Moreover, the culture filtrate of the endophytic fungus *Alternaria tenuissima* has also been used as an eco-friendly and cost-effective method for the synthesis of ZnO NPs (Abdelhakim et al., 2020). Phenols and Primary amine groups of active metabolites were mainly involved in the synthesis and capping of the ZnO NPs. Sharma et al. (2021); Abdelkader et al. (2022) illustrated the *Phanerochaete chrysosporium* and *A. niger* mediated synthesis of ZnO NPs. It has been noted that the carboxylic acids, phenolic acids, and amides in the enzymatic proteins of the fungal extract prevents the aggregation of ZnO NPs and helps in the stabilization of NPs.

No doubt microorganisms are extensively used for the synthesis of ZnO NPs, but still there are many drawbacks of this methodology. Firstly, isolation, selection and screening of microbes is time consuming. Secondly, mechanism of reduction and capping of NPs varies microbe to microbe. Most importantly, metal salt precursors sometimes get precipitated in the culture medium used for microbe growth. Furthermore, optimization of pH, temperature, and time for reaction set up is also crucial for the synthesis of ZnO nanoparticles. Therefore, in order to enhance the yield of ZnO NPs and the efficacy of this nano-factory further investigation in terms of detailed biochemical and molecular mechanism is required.

## 3 Role of ZnO NPs in biotic stress management

### 3.1 ZnO NPs as antimicrobial agents

**FIGURE 3:**
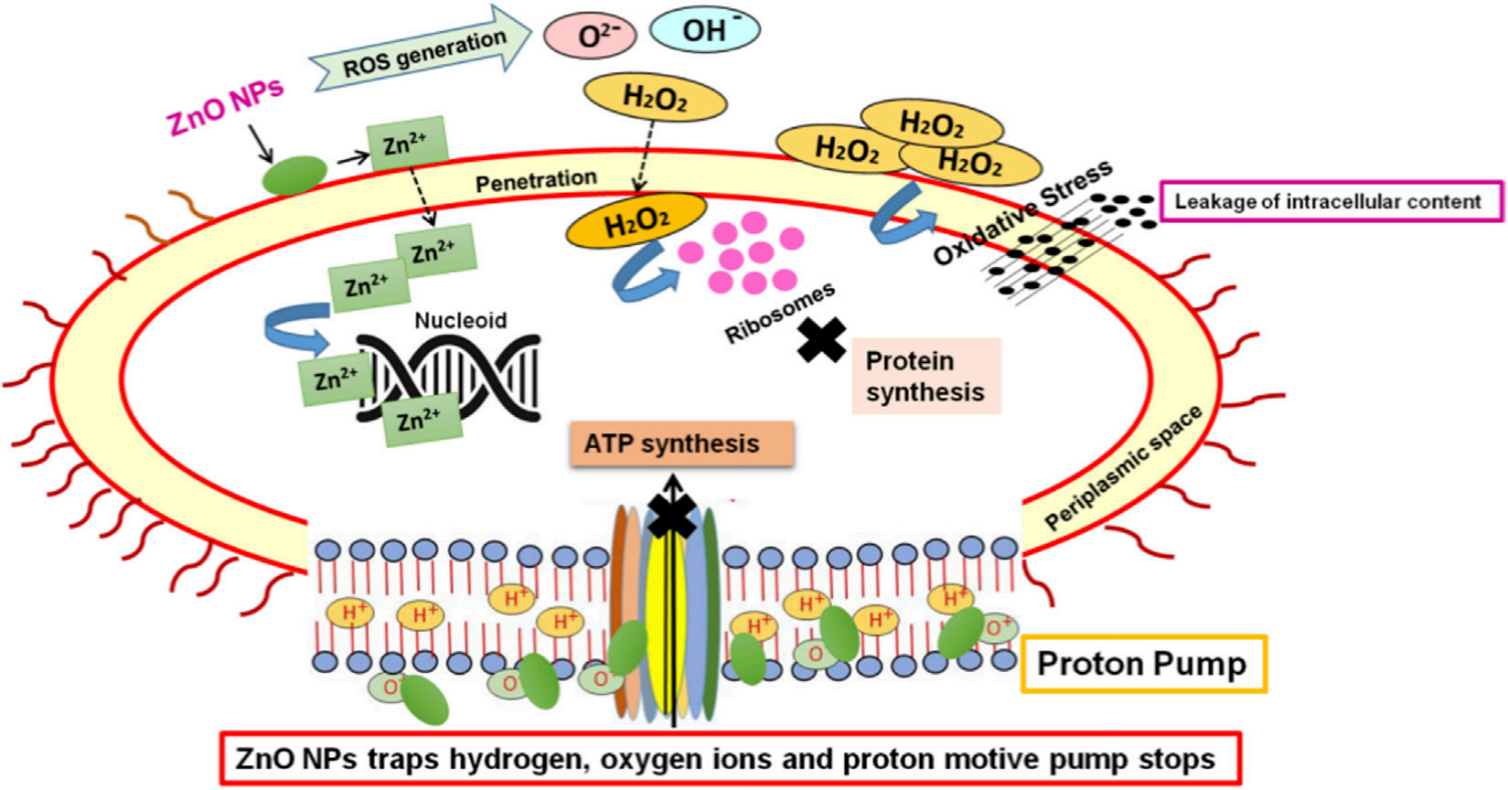
Mechanism of antibacterial acitivity of ZnO NPs.

ZnO NPs exhibit notable antifungal and antibacterial properties owing to their abrasive surface texture, smaller particle size, higher surface reactivity, and greater specific surface area (Singh et al., 2018). Previous investigations conducted by various research groups showed that metallic nanoparticles interfere with the cellular processes of the microbes and also cause cell immobilization (Janaki et al., 2015). Firstly, Zn^2+^ ions releases from ZnO NPs that inhibits the bacterial active transport, metabolism and enzyme activity. Secondly, ZnO NPs also interacts with the bacterial cell surface through electrostatic interaction, therefore stimulates the leakage of intracellular content. Thirdly, ZnO NPs also reacts with hydroxyl group and absorbs water that in turn induces the formation of ROS such as superoxide anion (O_2_^−^), hydroxyl ion (OH^−^), and hydrogen peroxide (H_2_O_2_). Out of all these ROS, H_2_O_2_ easily penetrates the bacterial cell membrane and damages the structure, integrity and function of biologically important biomolecules like ribosomes, lipids, proteins, nucleic acids, hence the bacterial cell dies (Mohd Yusof et al., 2019) (Figure 3).

Dimkpa et al. (2013) explored the antifungal efficacy of ZnO NPs suspension (500 mg Zn/l) against *Fusarium graminearum* pathogen, observing that the treatment with such higher level of Zn solution significantly reduced the 75% growth of the fungus on the MBA (mung bean agar) medium as compared to the control. The main reason behind the inhibitory action of ZnO NPs was the release of soluble Zn ions from the nanoparticles. Moreover, ZnO NPs synthesized by using the leaf extract of *Parthenium hysterophorus* L. also showed its antifungal activity against plant fungal pathogens including *Aspergillus flavus*, *Fusarium culmorum* and *A. niger*. It has been observed that 50 μg/mL concentration of 27 ± 5 nm size of ZnO NPs showed maximum zone of inhibition against *A. flavus* with inhibition diameter (24.66 ± 0.57 mm) (Rajiv et al., 2013). Kairyte et al. (2013) also explored the antibacterial activity of photo-activated ZnO NPs against *E. coli* O157:H7, *Listeria monocyto*genes ATCL3C 7644 and plant pathogenic fungus *Botrytis cinerea*. It has been observed that illumination of 1 × 10^−3^ M ZnO NPs with light (λ = 400 nm; 173 kJ/m^2^) significantly reduced the *E. coli* and *L. monocytogenes* population from 7log with 10 min–60 min incubation time. Meanwhile, treatment of fungus with 5 × 10^−3^ M concentration of photo-activated NPs reduced the mycelial growth by 58%. Interestingly, Narendhran and Sivaraj (2016) also studied the antifungal activity (against plant pathogens) of biogenic ZnO NPs (synthesized by using aqueous extract of *Lantana aculeata* Linn. Leaf). Results of well diffusion assay showed the highest zone of inhibition (21 ± 1.0 mm) against *A. niger* (MTCC: 10,180) followed by *Fusarium oxysporum* (MTCC: 3,326) (19 ± 1.0 mm) at 100 μg/mL concentration of ZnO NPs.

Anbukkarasi et al. (2015) investigated the antimicrobial activity of ZnO NPs against bacterial pathogens (*Bacillus subtilis*, *Streptococcus pneumonia*, *Staphylococcus epidermidis*, *Klebsiella pneumonia*, *Salmonella typhi,* and *E. coli*) and fungal pathogens (*A. niger,* and *Candida albicans*). Results indicate that 10 µg, 20µg, 40 µg, 60 µg and 80 µg concentration of ZnO NPs showed higher zone of growth inhibition against *S. epidermidis* (32 mm) followed by *E. coli* (31 mm), while no significant growth inhibition was observed against fungal pathogens. This underscores that ZnO NPs synthesized from *Emblica officinalis* is more effective against bacterial pathogens. Furthermore, antimicrobial activity of 20–25 mm size of 20, 40, 60, 80 and 100 μg/mL concentration of ZnO NPs was also studied against gram positive (*S. aureus*), gram negative bacteria (*Salmonella typhimurium*), and fungus (*A. flavus* and *A. fumigatus*). It has been noted that 40 μg/mL is the minimum inhibitory concentration (MIC) of ZnO NPs against both the bacterial pathogens. However, for fungal pathogens 60 μg/mL and 80 μg/mL concentration of ZnO NPs showed the excellent anti-fungal activity as compared to the 20, 40, and 100 μg/mL concentration (Navale et al., 2015). Another study also highlights the antibacterial activity of green synthesized ZnO NPs (using *Brassica oleraceae* leaf extract) against *E. coli*, *V. cholera*, *Clostridium botulinum*, *S. aureus,* and *B. subtilis*. It has been observed that highest zone of inhibition was observed in *S. aureus* (15 mm) followed by *B. subtilis* (14.5 mm), *E. coli* (13 mm), *C. botulinum* (10 mm), and *V. cholera* (9.5 mm) at 75 μg/mL concentration of ZnO NPs (Raj et al., 2015). Similar work also showed the antibacterial activity of green synthesized ZnO NPs (using aqueous peel extract of *A. vera*) against pathogenic bacteria, namely, *E. coli* (MTCC-41), *S. epidermidis* (MTCC-3382), *S. epidermidis* (MTCC-3382), *Klebsiella pneumoniae* (MTCC-3384), and fungi, *A. niger* (MTCC-404) and *Aspergillus oryzae* (MTCC-3107). Results revealed that synthesized ZnO NPs were only effective against *E. coli* (14 mm zone of inhibition) and *A. niger* (15 mm zone of inhibition). However no antimicrobial activity was observed in the case of other pathogens (Chaudhary et al., 2019) (Table 2).

**TABLE 2:** Anti-microbial activity of ZnO NPs over different pathogens.

Effective concentration or size of ZnO NPs	Synthesis method of ZnO NPs	Inhibitory effect of ZnO NPs	References
500 mg Zn/l	Commercially available ZnO NPs from Sigma-Aldrich	*Fusarium graminearum*	Dimkpa et al. (2013)
50 μg/mL	Leaf extract of *Parthenium hysterophorus* L	*Aspergillus flavus*, *Fusarium culmorum* and *Aspergillus niger*	Rajiv et al. (2013)
1 × 10^−3^ M	Commercially available ZnO NPs from Alfa Aesar	*Escherichia coli*, *Listeria monocyto*genes and *Botrytis cinerea*	Kairyte et al. (2013)
10 µg, 20 µg, 40 µg, 60 µg and 80 µg	Fruit methanol extract of *Emblica officinalis*	*Bacillus subtilis*, *Streptococcus pneumonia*, *Staphylococcus epidermidis*, *Klebsiella pneumonia*, *Salmonella typhi, Escherichia coli*, *Aspergillus niger* and *Candida albicans*	Anbukkarasi et al. (2015)
20, 40, and 100 μg/mL	Precipitation method	*Staphylococcus aureus*, *Salmonella typhimurium*, *Aspergillus flavus* and *A.fumigatus*	Navale et al. (2015)
6.25, 12.5, 25, 50 and 100 μg/mL	Leaf extract of *Brassica oleraceae*	*Escherichia coli*, *Vibrio cholera*, *Clostridium botulinum*, *Staphylococcus aureus,* and *Bacillus subtilis*	Raj et al. (2015)
100 μg/mL	Leaf aqueous extract of *Lantana aculeata* Linn	*Aspergillus niger* and *Fusarium oxysporum*	Narendhran and Sivaraj (2016)
-	Aqueous peel extract of *Aloe vera*	*Escherichia coli*, *S. epidermidis*, *Staphylococcus epidermidis*, *Klebsiella pneumoniae* and *Aspergillus niger*	Chaudhary et al. (2019)

Overall, we can conclude that ZnO NPs generally disturbs the metabolic processes of the microbes, generates oxidative stress, proton motive force over cell wall and oxidized the important cell wall structures which in turn lead to the death of the pathogens. This antimicrobial activity of these nanoparticles can be utilized to control fungal and bacterial pathogens in the agricultural fields.

### 3.2 Effect of ZnO NPs in disease mitigation

**FIGURE 4:**
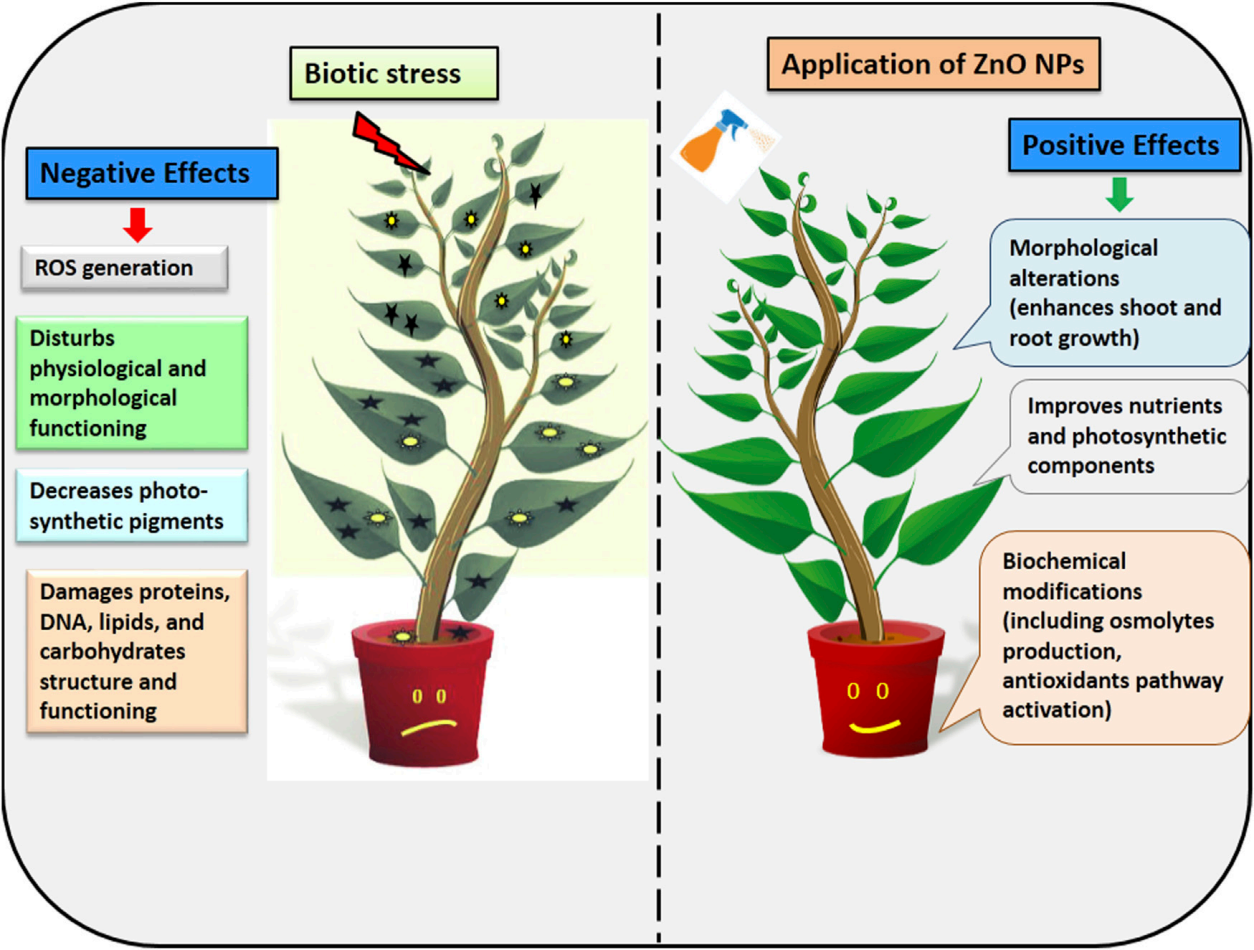
Application of ZnO NPs in improving plant growth during biotic stress.

Plants have evolved defense systems that trigger a range of signal transduction and perception pathways in order to protect themselves against biotic stress (Masri and Kiss, 2023). ZnO NPs also induce antioxidants machinery, osmolytes, and systemic resistance in plants in order to provide resistance against pathogens (Tortella et al., 2023) (Figure 4). These nanoparticles inhibit the pathogens by disturbing membrane potential, inducing DNA damage, cell damage, inducing ROS, cell cycle arrest, and by direct attachment to the cell surface (Khan et al., 2021a; Kalra et al., 2022).

A work carried out by Nandhini et al. (2019) evaluated the efficacy of green synthesized ZnO nanoparticles against downy mildew (caused by *Sclerospora graminicola*) disease in pearl millet. It has been observed that seed treatment and foliar spray with 50 ppm nanoparticles inhibited spore germination and reduced 35% disease incidence as compared to control plants. Furthermore, a significant increase in the activities of defense enzymes such as polyphenyl oxidase (PPO), peroxidase (POX), phenylalanine aminolyase (PAL), and lipoxygenase (LOX) were observed in the ZnO NPs treated plants. This clearly indicates that nanoparticles treatment provide disease protection by inducing systemic resistance in plants. Similarly, a research work conducted by Khan and Siddiqui (2021) showed the beneficial role of ZnO NPs (200 mg/L) in the disease management in *B. vulgaris* L. caused by *Meloidogyne incognita*, *Rhizoctonia solani*, and *Pectobacterium betavasculorum*. However, a significant reduction in the disease indices, nematode multiplication and root galling, along with an increase in the level of defensive antioxidants like superoxide dismutase (SOD), catalase (CAT), PAL and PPO were observed in the nanoparticles treated plants. Moreover, ZnO NPs also exhibit antiviral property and retard the growth of tobacco mosaic virus (ToMV) in tomato plants (Abdelkhalek and Al- Askar, 2020; Sofy et al., 2021). The foliar application of ZnO NPs (100 μg/mL) reduced viral accumulation and disease severity by 90.21% in affected tomato plants. Notably, the transcript level of genes including pathogenesis-related (PR-1), PAL, chalcone synthase (CHS), and peroxidase (POD) were also upregulated in the nanoparticles treated plants (Abdelkhalek and Al- Askar, 2020). Similarly in Sofy et al. (2021) research it has been noted that ZnO NPs (50 mg/L and 100 mg/L) application reduced the level of oxidative stress markers malondialdehyde (MDA), hydrogen peroxide (H_2_O_2_)and oxygen (O_2_) and promotes the activity of enzymatic (CAT, SOD, POX, ascorbate peroxidase (APX), glutathione reductase (GR), LOX, carbonic anhydrase (CA) and nitrate reductase (NR) and non-enzymatic antioxidants ascorbic acid (AsA), glutathione (GSH), proline and phenol).

Apart from viral infection, in the case of bacteria (*Pseudomonas syringae* pv. tomato, *Xanthomonas campestris* pv. *vesicatoria*, *Pectobacterium carotovorum* subsp. and *Ralstonia solanacearum*) and fungus (*F. oxysporum* f. sp. *lycopersici* and *Alternaria solani*) effected tomato plants, foliar and seed priming with ZnO NPs (0.20 mL^-1^) reduced disease incidence and improved photosynthetic pigments (total chlorophyll and carotenoids) and proline content in the diseased plants (Parveen and Siddiqui, 2021). Furthermore, antibacterial potential of ZnO NPs were also observed in the tomato plants. The results of concentration (0, 1, 4, and 8%) dependent application of ZnO NPs reduced the *R. solanacearum* population in the soil, disease severity and had increased shoot, root length, and fresh biomass (Khan et al., 2021b). Additionally, the ZnO NPs loaded hydrogel also showed beneficial role in protecting the pepper plant from pathogenic fungi (*F. oxysporum*). Application of hydrogel significantly reduced (77%) the wilt disease symptoms and also increased the chlorophyll, carotenoid content, osmolytes (soluble sugars, proteins, and proline), total phenolic content and the activity of POX and PPO in the diseased plants (Abdelaziz et al., 2021). Besides the hydrogel treatment results, similar results were also observed with ZnO NPs application or controlling *Fusarium* wilt disease in eggplant (Abdelaziz et al., 2022). Moreover, Farhana et al. (2022) research speculates that ZnO NPs also modulates the antioxidant machinery in chickpea for alleviating *Fusarium* wilt disease. It has been observed that seed priming significantly reduced the disease incidence by 90% and increased the quantities of sugars, phenol, flavonoids, total proteins and antioxidants (SOD, POD and CAT). Shams et al. (2023) also highlighted the efficacy of foliar spray of ZnO NPs against *Fusarium* wilt disease in cherry tomato. The results showed that 3000 ppm concentration of ZnO NPs reduced disease severity index (92.6%) and enhanced micronutrient (Mn, Zn, and Fe), chlorophyll content, and PAL enzyme activity. Another work carried out by Mazhar et al. (2023) showed that foliar application of ZnO NPs (10, 20, 30, and 40 mg/L) reduced the negative effect of blast fungus (*Magnaporthe oryzae*) and simultaneously promote the rice growth. ZnO NPs significantly decreased the lesion numbers, sporulation intensity, MDA, H_2_O_2_ level and also improved the total chlorophyll, activity of antioxidant enzymes like SOD, PAL, POX, and CAT.

However, the exact mechanism of action of ZnO NPs in reducing the pathogens attack is not clear yet and it varies from plant to plant. But overall from these cited literature studies, we can extrapolate that ZnO NPs usually reduces the bacterial, fungal, and viral infection by inducing systemic based resistance pathway, production of antioxidants, non-antioxidants enzymes, osmolytes (Table 3).

**TABLE 3:** Effect of ZnO NPs in ameliorating biotic stress in plants.

Disease/Pathogen	Plant	Concentration and mode of application of ZnO NPs	Synthesis method of ZnO NPs	Mechanism of mitigating disease	References
Downy Mildew (*Sclerospora graminicola*)	Pearl Millet	50 ppm	Aqueous extract of *Eclipta alba*	Plasmolysis and inhibition of spore germination. Enhanced activity of defense enzymes like PPO, POX, PAL and LOX	Nandhini et al. (2019)
*Meloidogyne incognita*, *Rhizoctonia solani, Pectobacterium betavasculorum*	Beet root	200 mg/L (Seed priming and Foliar spray)	Commercial available ZnO NPs from Sigma-Aldrich	Reduced nematode multiplication and root galling. Increased level of SOD, CAT, PAL and PPO	Khan and Siddiqui (2021)
Tobacco Mosaic virus-	Tomato	100 μg/mL (Foliar spray)	Aqueous leaf extract of *Mentha spicata*	90.21% reduction in viral accumulation. Upregulated activity of PR-1, PAL, CHS and POD	Abdelkhalek and Al- Askar (2020)
Tobacco Mosaic virus	Tomato	50 mg/L and 100 mg/L	-	Increased activity of CAT, SOD, POX, APX, GR, LOX, CA, NR, AsA and GSH	Sofy et al. (2021)
Bacterial and fungal pathogens	Tomato	0.20 mL^-1^ (Seed priming and Foliar spray)	Commercial available ZnO NPs from Sigma-Aldrich	Improved plant growth (27.8%–35.8% increase in shoot dry weight), total chlorophyll, carotenoids and proline content	Parveen and Siddiqui (2021)
*Ralstonia solanacearum*	Tomato	0, 1, 4, and 8%	Flower extract of *Matricaria chamomilla*	Decreased bacterial population in the soil. Increased shoot, root length, and fresh biomass of the treated plant	Khan et al. (2021a)
Wilt disease (*Fusarium oxysporum*)	Pepper	-	Chemically prepared ZnO hydrogel	Increased the chlorophyll, carotenoid, soluble sugars, proteins, proline content and activity of POX and PPO	Abdelaziz et al. (2021)
Wilt disease (*Fusarium oxtysporum*)	Egg plant	-	*Penicillium expansum* fungal filtrate extract	Reduced disease incidence. Improved plant height, root length, plant fresh biomass, chlorophyll a, chlorophyll b, total soluble protein and phenol content	Abdelaziz et al. (2022)
Wilt disease (*Fusarium oxysporum*)	Chickpea	Seed priming	Mycosynthesis (by using *Trichoderma harzianum* as a reducing and stabilizing agent)	Reduced the disease incidence by 90%. Increased the content of sugars, phenol, flavonoids and total proteins and activity of SOD, POD and CAT antioxidants were also enhanced	Farhana et al. (2022)
Wilt disease (*Fusarium oxysporum*)	Cherry tomato	3,000 ppm (Foliar spray)	Commercial available ZnO NPs from Nano-Gate Company, Cairo, Egypt	92.6% reduction in the disease severity. Enhanced Mn, Zn, Fe, chlorophyll content, and PAL enzyme activity	Shams et al. (2023)
Blast fungus (*Magnaporthe oryzae*)	Rice	10, 20, 30, and 40 mg/L (Foliar spray)	Commercial available ZnO NPs from Sigma Alderich	Decreased the lesion numbers, sporulation intensity, MDA and H_2_O_2_ level. Improved total chlorophyll and activity of SOD, PAL, POX, and CAT antioxidants	Mazhar et al. (2023)

## 4 Toxicity of ZnO nanoparticles

Recently ZnO NPs have been widely used in agricultural industries, but their toxicity is becoming a serious concern. There is very limited literature available on the toxicity of ZnO NPs in plants. Higher concentration of ZnO NPs exposure causes bioaccumulation in the plant tissue, exerts oxidative stress, nutrient imbalance, photosynthesis inhibition and causes lipid peroxidation of the plant cells leading to cell membrane damage (Amooaghaie et al., 2017; Song and Kim, 2020; Kang et al., 2024). As per a well-known mechanism that ZnO NPs can easily interacts with the plant defensive molecules like metallothionein and fully blocks their binding sites with Fe^2+^ and Cu^2+^ ions which in turns create ROS in the plants (Hou et al., 2018). It has been noted that the antimicrobial activity of ZnO NPs generally depends upon the concentration. It is important to evaluate the optimum concentration, dose and size of NPs to be used in agriculture (Shaw and Hossain, 2013).

The cytotoxicity of ZnO NPs, increase with the increase in the diameter of the particles. The toxic effect of ZnO NPs were also observed in maize plants at high doses, ranging from 800 mg/kg to 3,200 mg/kg, resulting in reduced growth of plants shoots and roots. Furthermore, mineral uptake by the shoots and roots, as well as the content of chlorophyll (a and b) and carotenoids also decreased (Liu et al., 2015). Furthermore, the concentrations between 500 mg and 1500 mg/L range were found to have detrimental effects on stem explant culture and seed germination of *Brassica nigra*. It has been observed that the presence of ZnO NPs in culture media inhibited the seed germination and reduced root length (Zafar et al., 2016). ZnO NPs of ∼85 nm with a concentration of 200–800 mg/L were found to induce cell death in root cells of *A. cepa, Vicia faba,* and *Nicotiana tabacum* plants. This induction was associated with increased levels of ROS production, resulting in DNA damage, H_2_O_2_ production, and lipid peroxidation (Ghosh et al., 2016). Moreover, *Hordeum vulgare* L. seedlings treated with 20 mg and 40 mg/kg concentrations of ZnO NPs decreased the level of chlorophyll content, SOD, APX, proline, and glutathione content as compared to control seedlings (Doğaroğlu and Köleli, 2017).

**FIGURE 5:**
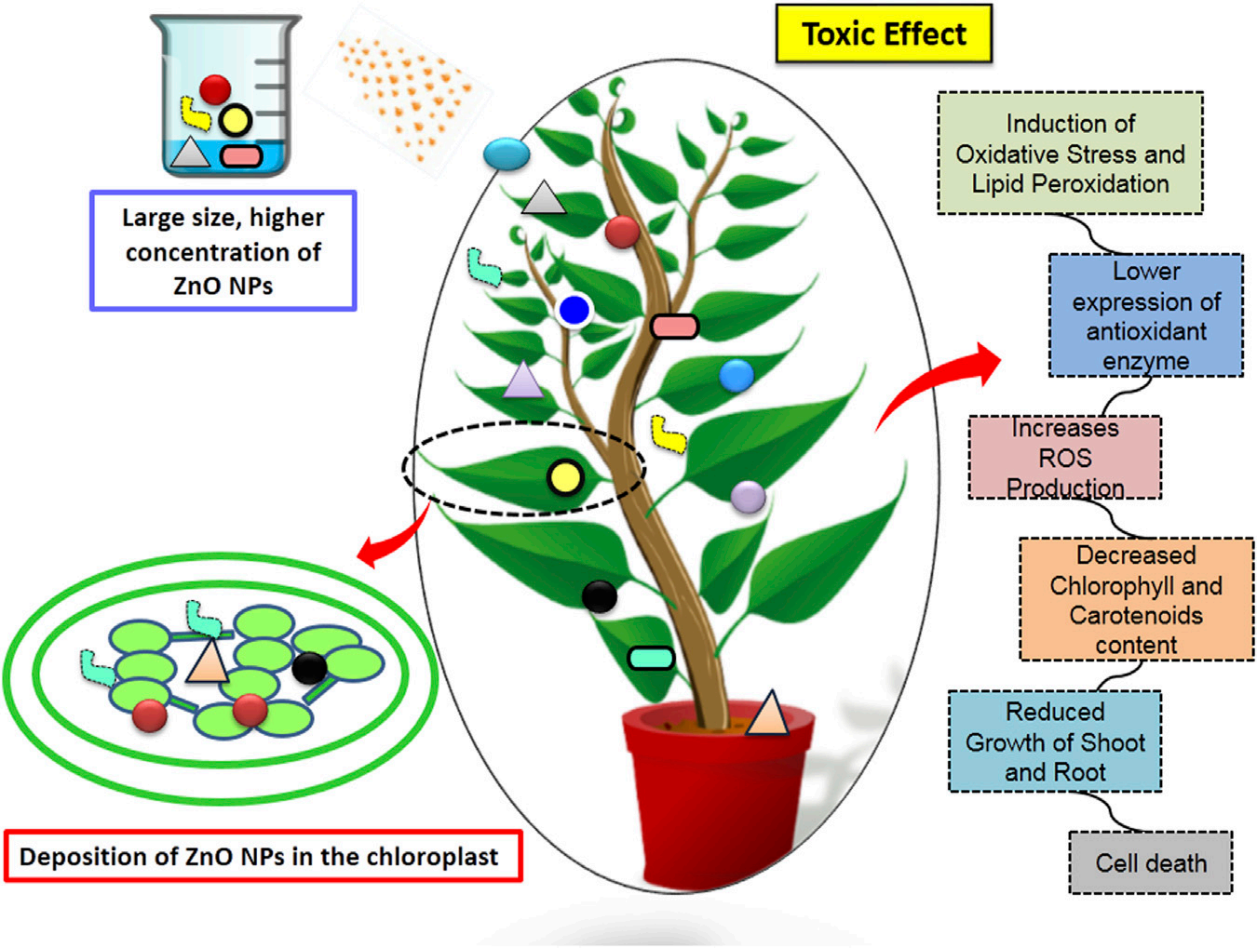
Toxic effect of large size ZnO NPs on plant health.

Similarly, in *Solanum lycopersicum* L. ZnO NPs (200mg–800 mg dm^-3^) negatively affect the growth of plant shoot, root and reduced the content of chlorophyll a and b. Moreover, gene expression analysis indicated downregulation of key photosynthesis genes such as *sedoheptulose-1,7-bisphosphatase* (*SBPASE*) and *fructose-1,6-bisphosphatase* (*FBPASE*) and chlorophyll synthesis genes including *chlorophyllide a oxygenase* (*CAO*), *chlorophyll synthase* (*CHLG*), *magnesium-protoporphyrin IX monomethylester* [oxidative] *cyclase* (*CRD1*), *magnesium-chelatase subunit ChlI* (*CHL1*), *protoporphyrinogen oxidase* (*HEMG*), *5-aminolevulinate dehydratase* (*HEMB*), and *porphobilinogen deaminase* (*HEMC*) when plants were exposed to high concentrations of toxic ZnO NPs (Wang et al., 2018). Additionally, another research work conducted by Czyżowska and Barbasz (2019) on the callus of wheat varieties reported that applying ZnO NPs (130 nm diameter and 12 mg dm^-3^ concentration) reduced the activity of SOD and generates oxidative stress (Table 4; Figure 5). Furthermore, their research demonstrated that the harmful effects of ZnO NPs on plants were also heightened in the presence of UV radiation.

**TABLE 4:** Toxic effect of ZnO NPs over physiological, biochemical and molecular functioning of different plants.

Plant	Concentration of ZnO NPs	Mode of application of ZnO NPs	Toxic effect	References
Maize	800 mg/kg - 3,200 mg/kg	Direct mixing of ZnO powder in the soil	Reduced mineral uptake, chlorophyll and carotenoid content	Liu et al. (2015)
Mustard	500mg–1500 mg/L	ZnO NPs were supplemented in the culture medium	Inhibited seed germination and stem explant culture growth	Zafar et al. (2016)
Onion, Tobacco and Faba beans	200–800 mg/L	Hydroponic treatment	Induced root cells death and DNA damage. Increased production of ROS and H_2_O_2_	Ghosh et al. (2016)
Barley	20 mg and 40 mg/kg	Seed treatment for 7 days in the dark at 25°C	Decreased chlorophyll, SOD, APX, proline, and glutathione content	Doğaroğlu and Köleli (2017)
Tomato	200mg–800 mg dm^-3^	Three-week old seedlings were treated with ZnO NPs suspension	Reduced shoot and root growth, chlorophyll a and chlorophyll b content. Downregulated the expression of SBPASE, FBPASE, CAO, CHLG, CRD1, CHL1, HEMG, HEMB and HEMC photosynthesis genes	Wang et al. (2018)
Wheat	12 mg dm^-3^	Callus cells	Increased MDA content and reduced the activity of SOD and POX antioxidant activity	Czyżowska and Barbasz (2019)

In summary, toxicity of these nanoparticles can be overcome by optimizing the size and shape of the nanoparticles. Thus, by keeping all these parameters in consideration toxicity of nanoparticles can be reduced.

## 5 Conclusion

In conclusion, the pressing issue of biotic stress in agriculture, compounded by global warming and shifting climate patterns, needs new and sustainable crop management measures. This review explored into the possibility of zinc oxide nanoparticles (ZnO NPs) as a viable solution to biotic stress due to their antibacterial characteristics and role as nano-fertilizers. The biological synthesis of ZnO NPs, which utilizes plants and microorganisms, provides an environmentally friendly and possibly scalable approach to produce these nanoparticles.

ZnO NPs are highly effective in treating a variety of pathogens, including bacteria, fungi, viruses, and insects, by affecting their physiological and molecular processes. Despite the fact that these particles have numerous applications on plants, there have been reports of their possible toxicity. The potential toxicity of ZnO nanoparticles on plants can be reduced by employing strategies such as dosing optimization, controlled release systems, nanoparticle surface modification, and combination with other agents can be used to ensure effective biotic stress mitigation while minimizing adverse effects. Additionally, using targeted delivery and combining with sustainable methods improves safety and efficacy. However, the use of these nanoparticles in agriculture is still in its early stages, with numerous problems that must be addressed. These include studying the mechanisms of action of ZnO NPs under various biotic stress circumstances, optimizing synthesis methods, defining the most effective nanoparticle size and shape, and establishing safe concentration limits to avoid plant toxicity. Future research should focus on resolving these limitations in order to fully utilize ZnO NPs in agricultural applications. Rigorous testing and regulation will be required to ensure their safety and sustainability.

To summarize, while ZnO NPs provide intriguing options for biotic stress alleviation, their use should be addressed with caution. Comprehensive research and careful implementation are required to maximize the advantages while limiting the dangers. By improving our understanding and application of ZnO NPs, we can help to build a more sustainable and resilient agricultural sector, thereby aiding worldwide efforts to secure food production and promote environmental sustainability.
